# An investigation of the measurement properties of the de Morton Mobility Index for measuring mobility capacity in hospital patients with Parkinson’s disease

**DOI:** 10.1177/0269215520966472

**Published:** 2020-11-11

**Authors:** Tobias Braun, Detlef Marks, Christian Thiel, Alexandra Menig, Christian Grüneberg

**Affiliations:** 1 Division of Physiotherapy, Department of Applied Health Sciences, Hochschule für Gesundheit (University of Applied Sciences), Bochum, Germany; 2 Physiotherapy Department, Rehaklinik Zihlschlacht, Zihlschlacht, Thurgau, Switzerland; 3 Faculty of Sports Science, Training and Exercise Science, Ruhr-University Bochum, Bochum, Germany; 4 Occupational Therapy Department, Rehaklinik Zihlschlacht, Zihlschlacht, Switzerland

**Keywords:** Parkinson’s disease, neurological rehabilitation, outcome assessment, mobility limitation, reproducibility of results

## Abstract

**Objective:**

To examine the measurement properties of the de Morton Mobility Index (DEMMI), a performance-based clinical outcome assessment of mobility capacity, in hospital patients with Parkinson’s disease.

**Design:**

Cross-sectional study.

**Participants:**

Hospital patients with Parkinson’s disease.

**Main outcome measure(s):**

Structural validity and unidimensionality (Rasch analysis), construct validity, internal consistency reliability, and inter-rater reliability of the de Morton Mobility Index (scale range: 0–100 points) were established. The minimal detectable change, the 95% limits of agreement and possible floor and ceiling effects were calculated to indicate interpretability.

**Results:**

We analysed validity (*n* = 100; mean age: 70 years; 71% male) and reliability (*n* = 47; mean age: 71 years; 68% male) in two samples. The mean Hoehn and Yahr stage was 3.2 and the mean disease duration was 12 years in both samples. Rasch analysis indicated unidimensionality with an overall fit to the model (chi-square = 21.49, *P* = 0.122). Seventy-three percent of hypotheses on construct validity were confirmed. Internal consistency reliability (Cronbach’s alpha = 0.91) and inter-rater reliability (intraclass correlation coefficient = 0.88; 95% confidence interval: 0.80 to 0.93) were sufficient. The minimal detectable change with 90% confidence was 17.5 points and the limits of agreement were 31%. No floor or ceiling effects were observed. The mean administration time was 6.6 minutes.

**Conclusion:**

This study provides evidence of unidimensionality, sufficient internal consistency reliability, inter-rater reliability, construct validity, and feasibility of the de Morton Mobility Index in hospital patients with Parkinson’s disease.

**Trial registration:**

German Clinical Trials Register (DRKS00004681). Registered May 6, 2013.

## Introduction

Mobility assessment in people with Parkinson’s disease is advised in clinical practice guidelines,^1,2^ especially to monitor disease progression, effectiveness of medication changes, and efficacy of pharmacological and rehabilitation interventions, among others. The European physiotherapy guideline for Parkinson’s disease recommends a set of measurement instruments to assess balance and gait in people with Parkinson’s disease.^1^ However, no “gold standard” exists for the assessment of the above-mentioned two constructs and “mobility”.^3,4^ Moreover, most of the available and recommended tools measure only one aspect of mobility, as defined by the World Health Organization,^5^ such as gait speed or ambulation, sit-to-stand transfers, or balance.

The de Morton Mobility Index (short form: DEMMI) is a performance-based, unidimensional, interval-level, and feasible outcome measure of older people’s mobility capacity. This clinical outcome assessment was developed using the Rasch model,^6,7^ and there is strong evidence for high psychometric quality in various health care settings and clinical populations.^8–15^ The de Morton Mobility Index form consists of one paper sheet and can be administered within 10 minutes without special equipment.^8,11^

Johnston et al.^16^ compared the de Morton Mobility Index with other commonly used activity-related measures of mobility and balance in a population of community-dwelling people with Parkinson’s disease visiting an outpatient community rehabilitation facility. Participants presented with a mild-to-moderate disease severity (modified Hoehn and Yahr staging between 2 and 3). The study provides first evidence for the de Morton Mobility Index as a promising measure of mobility capacity in people with Parkinson’s disease since a Rasch analysis confirmed unidimensionality.^16^ However, the authors did not examine reliability, measurement error or interpretability. Furthermore, validity of the de Morton Mobility Index in more severely affected hospital inpatients with Parkinson’s disease remained unclear.

The aim of this study was to examine the psychometric properties of the de Morton Mobility Index in people with Parkinson’s disease visiting a rehabilitation hospital by the means of modern methods of latent trait theory (Rasch analysis)^6,7^ and methods of classical test theory.

## Methods

Reporting of this study was informed by the STrengthening the Reporting of OBservational studies in Epidemiology (STROBE) guideline for observational studies,^17^ the Guidelines for Reporting Reliability and Agreement Studies (GRRAS)^18^ guideline for reliability studies, and criteria of the COnsensus-based Standards for the selection of health Measurement Instruments (COSMIN) risk of bias checklist.^19^

We performed a cross-sectional study on the psychometric properties of the de Morton Mobility Index in neurorehabilitation. This study was approved by the Local Committee for Ethics in Medical Research (Ethikkommission Kanton Thurgau, Switzerland; application 2013/13), performed in accordance with the Helsinki Declaration of 1975 (as revised in 2013), was registered *a priori* (German Clinical Trials Register: DRKS00004681), and all participants gave written informed consent. Here, we report on structural and construct validity, internal consistency, inter-rater reliability, measurement error, interpretability and feasibility of the de Morton Mobility Index in a sub-sample of participants with Parkinson’s disease.

**Figure 1.**
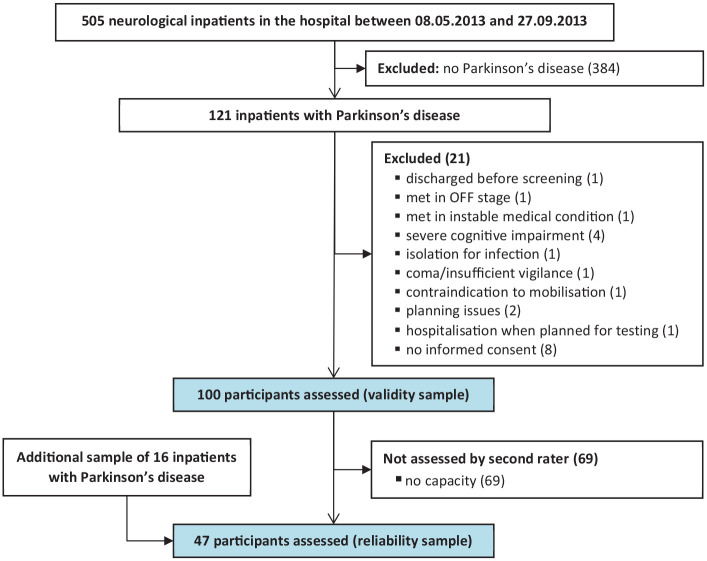
Flow chart of study participants.

The study was conducted at the Rehaklinik Zihlschlacht, a neurological rehabilitation center in Switzerland. Patients with Parkinson’s disease (all stages of disease severity) are typically referred to the center by acute hospitals, neurologist consultants, or general practitioners located in the eastern and central parts of Switzerland. During inpatient rehabilitation, participants received multimodal, interprofessional and intensive rehabilitation, according to clinical practice guidelines.^1^ The study sample consisted of all inpatients present on May 8, 2013 or entering the rehabilitation center consecutively in the following 20 weeks. The inclusion criteria were: (1) idiopathic Parkinson’s disease (ICD-10 code: G.20.0) and (2) ⩾18 years of age. The main exclusion criteria were severe cognitive impairment and a contraindication for mobilization (all criteria listed in Figure 1).

Eligible participants were examined by the primary investigator (TB), a physical therapist with five years of clinical and academic working experience. The primary investigator was blinded toward the mobility status of the participants. In a single session of 30 to 45 minutes, the de Morton Mobility Index and a comprehensive set of motor function measures were performed in a standardized order, within seven days after hospital admission if possible (baseline). Socio-demographic data were sourced from the medical records. The Hoehn and Yahr stage (range: 0–5)^20^ was rated by the hospital neurologist. Higher stages indicate higher disease severity.

Inter-rater reliability was examined between two physiotherapists, the primary investigator (TB) and a second rater (DM) with 25 years of clinical working experience. Both raters had substantial practical and theoretical experience and knowledge in the use of outcome measures, and the de Morton Mobility Index in particular, as both raters had previously administered the measure approximately 100 times in clinical care.

The second rater performed the de Morton Mobility Index independently in a convenient sub-sample (reliability sample). Selection was mainly based on the availability of the second rater (temporal resources) and on participants’ consent to perform a second assessment. Both measures were performed within two days. To create a stable retest situation, participants were excluded if they reported a change in their physical and mental condition (e.g. fatigue or pain) with respect to the first session. The test environment (patient’s room) was similar for both sessions. Both measures (baseline and retest) were performed in the patient’s ON stage. Both raters were blinded to the results of the other. While randomization of the order of assessors was not possible due to the clinical procedures, we tried to balance the number of participants each assessor visited first. Therefore, we aimed for half of the participants to be assessed by the principal investigator first.

Within the initial recruitment period (20 weeks), we did not reach a “good” sample size of 50 participants for the reliability analysis.^21,22^ Hence, we set up a second recruitment period and screened all present and incoming patients over a period of nine consecutive days. The inclusion criteria were similar. The included sample of convenience was used for the inter-rater reliability analysis only.

Participants were assessed with the de Morton Mobility Index and a set of functional assessments (listed in Table 1). The de Morton Mobility Index is a performance-based bedside assessment of mobility capacity, consisting of 15 hierarchical mobility items.^8^ The patient is asked to perform functional tasks associated with bed and chair mobility, ambulation, static balance, and dynamic balance. The items are rated with 2-or 3-point response options, resulting in a maximum ordinal score of 19 points. This raw score can be transformed into a total interval score of 0 to 100 points, with higher scores indicating a higher level of mobility. We used a validated German language translation.^11,12,23^

**Table 1.** Construct validity of the de Morton Mobility Index (*n* = 100): Hypotheses on construct validity; scale range, construct and results of comparison measurement instruments; magnitude of correlation or statistical significance of difference; and confirmation or rejection of hypotheses.

No.	Hypotheses	Comparison measurement instrument			Observed correlation with DEMMI (Spearman’s correlation)		Hypothesis confirmed
		Measurement instrument	Construct	Mean ± SD (range) or median (IQR)	rho	95% CI	
1	A correlation of >0.7 was expected between the DEMMI and other broad measures of mobility, ambulation, walking endurance and balance	POMA, 0–28 points	Mobility	19 ± 8 (0–28)	0.91	0.87 to 0.94	Yes
2		Timed Up and Go test (*n* = 86), sec^1^	Mobility	15 ± 12 (5–76)	0.71	0.58 to 0.80	Yes
3		FIM mobility sub-scale (*n* = 95), 5–35 points	Mobility	23 ± 6 (5–33)	0.71	0.60 to 0.80	Yes
4		FAC, 0–5 points	Ambulation	5 (4–5)	0.80	0.72 to 0.86	Yes
5		6-minute walk test (*n* = 91), meter	Walking endurance	340 ± 153 (44–620)	0.76	0.66 to 0.83	Yes
6		Berg Balance Scale, 0–56 points	Balance	40 ± 16 (0–56)	0.91	0.87 to 0.94	Yes
7	A moderate correlation (0.5<rho ⩽ 0.7) was expected between the DEMMI and other single-component mobility scales, measures of motor functioning and freezing of gait	10-meter gait speed (*n* = 92), m/sec	Gait speed	1.14 ± 0.47 (0.15–2.00)	0.64	0.50 to 0.75	Yes
8		5x chair rise test (*n* = 65), sec^1^	Lower limb strength	14 ± 4 (7–26)	0.24	0 to 0.45	No
9		UPDRS Part II (*n* = 83), 0–52 points^1^	Activities of daily living	16 ± 6 (3–36)	0.49	0.31 to 0.64	No
10		UPDRS Part III (*n* = 83), 0–108 points^1^	Motor functioning	31 ± 11 (6–65)	0.35	0.15 to 0.52	No
11		FIM total (*n* = 95), 18–126 points	Functional independence	85 ± 19 (25–123)	0.63	0.49 to 0.74	Yes
12		FOGQ (*n* = 91), 0–24 points^1^	Freezing of gait	10 ± 6 (0–22)	0.40	0.21 to 0.56	No
	Hypotheses	Observed mean DEMMI scores (points) according to clinical groups			Statistical significance (Mann-Whitney U test, 1-fold)		Hypothesis confirmed
		Clinical groups	DEMMI mean score				
13	A statistically significant mean difference between ambulatory (FAC ⩾ 3; *n* = 93) participants walking without versus participants walking with a walking aid.	No walking aid (*n* = 47)	87 ± 14 (24–100)		U = 170; *P* < 0.01		Yes
		Walking aid (*n* = 46)	54 ± 13 (15–74)				
13	A statistically significant mean difference between independently ambulatory (FAC ⩾ 4) versus dependently ambulatory/non-ambulatory (FAC ⩽ 3) participants.	Independent walkers (*n* = 77)	71 ± 15 (33–100)		U = 118; *P* < 0.01		Yes
		Dependent/non-ambulatory (*n* = 23)	36 ± 20 (0–67)				
15	A statistically significant mean difference between participants with a mild-moderate (HY 1–3) and participants with a severe (HY 4–5) symptoms of Parkinson’s disease.	HY stage 1–3 (*n* = 63)	72 ± 15 (33–100)		U = 411; *P* < 0.01		Yes
		HY stage 4–5 (*n* = 37)	48 ± 23 (0–100)				

SD: standard deviation; IQR: interquartile-range; CI: confidence interval; DEMMI: de Morton Mobility Index; POMA: Performance Oriented Mobility Assessment; FIM: functional independence measure; FAC: functional ambulation categories; UPDRS: Unified Parkinson Disease Rating Scale; FOGQ: Freezing of Gait Questionnaire; HY: Hoehn and Yahr scale.

1 indicates hypothesis of a negative correlation.

Detailed descriptions of the assessment procedures and the comparator instruments are given in Supplemental File 1. Table 1 provides an overview of the scale width and constructs measured by the comparator instruments.

Data were analysed using SPSS version 21.0 and Microsoft Excel (Professional Plus 2016) for all analyses except the Rasch analysis, which was completed using RUMM2030 version 5.1 software. Descriptive statistics were used to present sample characteristics. Interval-based data were examined for normal distribution with the Shapiro-Wilk test of normality and by visual inspection of the related histograms and p-p-plots. As the de Morton Mobility Index scores were not normally distributed (*P* < 0.001), only non-parametric statistics were applied. A significance value of 5% was used throughout.

The de Morton Mobility Index was developed based on the Rasch model^6^ in older acute medical patients.^8^ Data fitted the model in various other medical conditions^12,13,15,24^ and in people with Parkinson’s disease living in the community.^16^ The Rasch model is a probabilistic model that asserts that item response is a logistic function of item difficulty and person ability.^6^

We initially performed a Rasch analysis to check if the unidimensionality, hierarchical order, internal validity, and logistic item structure of the de Morton Mobility Index remain valid in people with Parkinson’s disease. Overall fit of data to the model was deemed acceptable if a set of criteria was fulfilled (Supplemental File 2). Full details of the Rasch analysis process are given elsewhere.^7,25^ Reporting followed established recommendations.^7^

A target sample size of at least 100 was set for this study to provide 95% confidence within ±0.5 logits.^26^ The unrestricted (partial credit) Rasch polytomous model was used with a conditional pair-wise parameter estimation.

Construct validity was assessed by following the methodological approach of hypotheses testing, since there is no “gold standard” to measure the construct of mobility capacity.^21,22^ We used the other functional outcomes as well as participants’ clinical information to assess the construct validity of the de Morton Mobility Index. Aspects of convergent and known-groups validity were used to formulate 15 hypotheses (H1 to H15).^22,27^ All hypotheses were formulated *a priori*, based on existing literature and clinical expertise of clinicians and the research team.^8,12,13,16,23^ Formulated and shortened versions of all hypotheses are presented in Supplemental File 2 and Table 1, respectively. Detailed information on the statistical analyses and interpretations of hypotheses testing are given in Supplemental File 2. A target sample size of at least 100 participants was set.^28^

Cronbach’s alpha, a measure of internal consistency for a unidimensional scale, was derived from the validity sample because of its large sample size.^22^ An outcome between 0.7 and 0.95 was considered acceptable.^22^

Inter-rater reliability was examined using the intra-class correlation coefficient (ICC) model 2.1 (two-way random effects model; ICC_AGREEMENT_).^27^ ICC ⩾0.7 was deemed acceptable.^22^ The standard error of measurement (SEM_AGREEMENT_) was calculated^27^ and deemed satisfactory if it was ⩽10% of the total scale range (100 points).^29^ The absolute and relative agreement between both raters per item was calculated as percentage (%) and as weighted kappa with linear weights (ƙ), respectively.^27^ Agreement per item equal to or above 70% and ƙ ⩾0.70 were considered acceptable.^22^ Additional information on reliability statistics is given in Supplemental File 2.

The method of Bland and Altman was used to illustrate agreement between the two raters.^30^ The minimal detectable change (MDC) values with 90% and 95% confidence were calculated.^31^ A floor or ceiling effect was considered if ⩾15% of the participants scored the highest or lowest possible de Morton Mobility Index score.^22^ Supplemental File 2 provides more information on the statistical methods.

To assess feasibility, we calculated the mean administration time for the de Morton Mobility Index in minutes, and related the administration time to the functional status of the participants. We documented any adverse event, such as falls, reports of pain, untypical and severe changes in muscle tone, or significant fatigue.

## Results

One-hundred inpatients with Parkinson’s disease were assessed within the first recruitment period for the validity sample. For the inter-rater reliability analysis, 47 participants were included (flow of the participants in Figure 1; participants’ demographics in Table 2).

**Table 2.** Baseline characteristics of the participants with Parkinson’s disease by sample.

Characteristic	Validity sample (*n* = 100)	Reliability sample (*n* = 47)
Age in years	70 ± 9 (34–90)	71 ± 10 (34–85)
Mini Mental State Examination, points	25 ± 5 (5–30) (*n* = 98)	25 ± 4 (12–30) (*n* = 46)
Male gender	71 (71%)	32 (68%)
Hoehn & Yahr stage	3.2 ± 0.8 (1–5)	3.2 ± 0.9 (1–5)
Stage I	4 (4%)	3 (6%)
Stage II	7 (7%)	2 (4%)
Stage III	52 (52%)	23 (49%)
Stage IV	35 (35%)	18 (38%)
Stage V	2 (2%)	1 (2%)
Unified Parkinson Disease Rating Scale part II: activities of daily living, 0–52 points	16 ± 6 (3–36) (*n* = 83)	15 ± 6 (3–33) (*n* = 27)
Unified Parkinson Disease Rating Scale part III: motor evaluation, 0–108 points	31 ± 11 (6–65) (*n* = 83)	30 ± 8 (17–47) (*n* = 27)
Disease duration, years		
Mean, SD (range)	12 ± 7 (1–44)	12 ± 9 (1–44)
Median (IQR)	10 (7–16)	10 (6–16)
Time since admission at baseline assessment in days		
Mean, SD (range)	5 ± 7 (1–50)	8 ± 8 (1–35)
Median (IQR)	3 (2–7)	6 (3–10)
Walking aid		
None	47 (47%)	22 (47%)
Rollator/walker	35 (35%)	17 (36%)
One cane/stick	6 (6%)	2 (4%)
Two crutches/walking sticks	5 (5%)	4 (9%)
Not ambulatory/wheelchair	7 (7%)	2 (4%)
de Morton Mobility Index, points	63 ± 22 (0–100)	64 ± 23 (0–100)

Values are mean ± standard deviation (range), median (interquartile range) or absolute numbers (%).

The mean (SD) age of the participants was 70 (9) years, most showed moderate (*n* = 52; 52%) or severe (*n* = 37; 37%) symptoms according to Hoehn and Yahr staging (stage 3 and 4, respectively), and 53% (*n* = 53) of the participants were not able to walk or needed a walking aid. Eighty-one percent of the participants were assessed within seven days after hospital admission, and 95% within 14 days. None of the de Morton Mobility Index assessments in this study contained missing items (distributions of scores illustrated in Supplemental Figure 1). Table 1 includes the mobility related outcomes for all comparator instruments.

**Figure 2.**
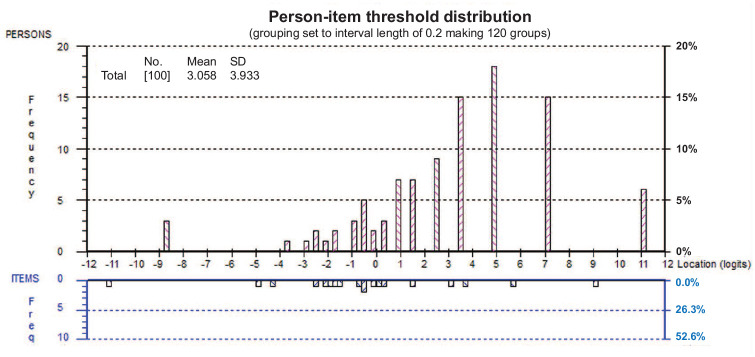
Person-item distribution of the de Morton Mobility Index.

Rasch analysis was performed on the complete de Morton Mobility Index item sets of 100 participants and on the complete 15-item scale. Overall fit to the model was achieved with a non-significant chi-square value (21.49, degrees of freedom = 15, *P* = 0.122). There were no mis-fitting persons and no mis-fitting items as all person-fit and item-fit residuals were within ±2.5. The mean item-fit residual and person-fit residuals were −0.35 ± 0.34 and −0.14 ± 0.16, respectively. There were no disordered thresholds, indicating that the responses to the items were consistent with the metric estimate of the underlying construct of mobility. The Person Separation Index was 0.86. Unidimensionality was further confirmed with 2.2% (95% confidence interval (CI): 0 to 6.7) significant independent *t*-tests at the person level. Data were free of local dependency. There was no Differential Item Functioning by sex, age, or cognitive impairment, indicating that none of these factors caused item bias. Overall, the respondents exhibited a higher level of mobility (3.1 logits) than the scale average (0.0 logits) (Figure 2).

**Figure 3.**
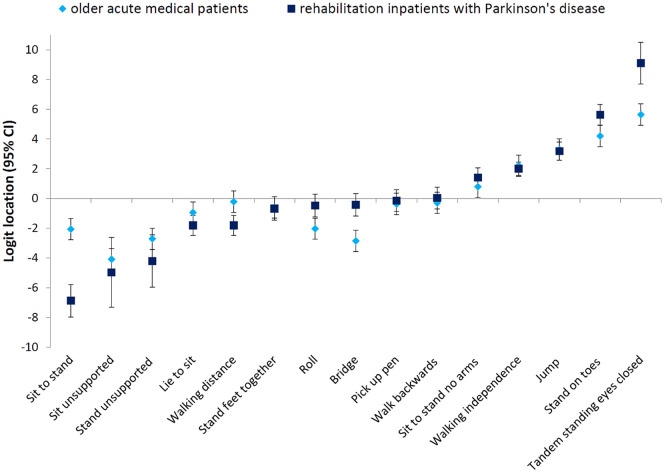
Item logit location (with 95% confidence intervals) and item hierarchy of difficulty for the sample of rehabilitation inpatients with Parkinson’s disease and the original older acute geriatric sample de Morton Mobility Index data.^8^ A high positive logit location (e.g. standing on toes) indicates harder item difficulty compared to a negative logit location (e.g. roll). Deviation from the original hierarchy is indicated by non-overlapping 95% confidence bands.

The item hierarchy of the de Morton Mobility Index in the sample of inpatients with Parkinson’s disease compared to that of the development sample (geriatric inpatients)^8^ is illustrated in Figure 3. A high positive logit location (e.g. tandem standing eyes closed) indicates harder item difficulty compared to a negative logit location (e.g. sit to stand). Deviations from the original item hierarchy are indicated by non-overlapping 95% confidence bands in five items.

The analyses on construct validity showed that 11 (73%) of the 15 a priori stated hypotheses about correlations of the de Morton Mobility Index with other clinical measures and known-group differences were confirmed (Table 1).

Cronbach’s alpha of the de Morton Mobility Index was 0.91, indicating sufficient internal consistency reliability.

Rater 1 (TB) administered the first de Morton Mobility Index measure in 32 of 47 participants (68%). Both measures were performed on the same day in 29 participants (62%), and within two days in 18 participants (38%). No statistically significant mean differences were observed in the scores between both assessors (0.3 points; 95% CI: −2.9 to 3.4; *P* = 0.87). The ICC_AGREEMENT_ was 0.88 (95% CI: 0.80 to 0.93).

The SEM_AGREEMENT_ was 7.5 points and considered acceptable (7.5% of the total scale range of the de Morton Mobility Index).

The absolute and relative agreement per item are presented in the table in Supplemental File 3. There was no de Morton Mobility Index item with absolute agreement <70% (range from 72% to 100%; Supplemental Figure 2), but 10 items with ƙ <0.7 (range from 0.30 to 1.0).

**Figure 4.**
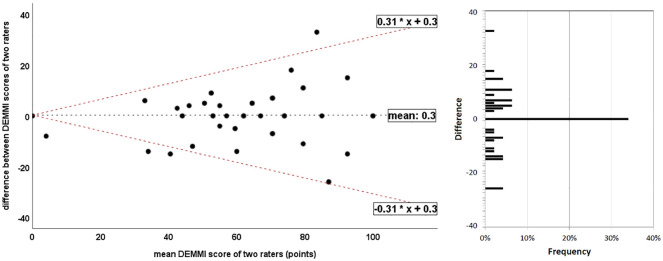
Bland-Altman plot of de Morton Mobility Index (DEMMI) scores by two raters. The *x*-axis represents the mean sores of the raters and the *y*-axis represents the difference between the raters. The dotted black line represents the mean difference between both measures; dotted red lines represent the 95% upper and lower limits of agreement. The bar chart on the right side illustrates the frequency of differences between the two raters.

The Bland-Altman plot is illustrated in Figure 4. Data were heteroscedastic (τ = 0.20) and differences were not normally distributed (*P* = 0.03). The 95% limits of agreement were 0.31X+0.3 and −0.31X+0.3, respectively, with X denoting the mean score.

The minimal detectable change values were 17.5 (MDC_90_) and 20.9 (MDC_95_) points, respectively.

Supplemental Figure 1 illustrates that neither absolute floor nor ceiling effects occurred, since three participants (3%) scored 0 de Morton Mobility Index points and six participants (6%) scored 100 points.

**Figure 5.**
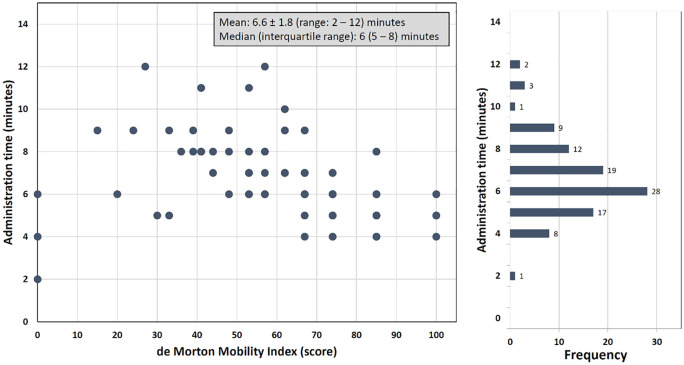
Administration time of the de Morton Mobility Index according to scale range.

The mean administration time of 100 de Morton Mobility Index assessments was 6.6 ± 1.8 minutes (range: 2–12) (Figure 5). In non-ambulant or dependent walkers (*n* = 23) and independent walkers (*n* = 77), the de Morton Mobility Index administration time was 8.2 ± 2.5 and 6.2 ± 1.3 minutes, respectively. No adverse events occurred in any de Morton Mobility Index assessment.

## Discussion

The Rasch analysis confirmed structural validity in terms of unidimensionality, hierarchical order, and logistic item structure of the de Morton Mobility Index, and these results have also been reported for various other geriatric^8,12,13,24^ and neurological populations.^15,16^ Notably, evidence of unidimensionality is highly important, since clinicians and researchers can be confident that the de Morton Mobility Index measures one single underlying construct (mobility capacity) in hospital patients with Parkinson’s disease.

The de Morton Mobility Index showed moderate to strong correlations with other validated and established measures of mobility, balance and ambulation, indicating good construct validity. However, four (27%) of the a priori stated hypotheses were not confirmed. These hypotheses concern correlations between the de Morton Mobility Index and measures of lower limb strength (chair rise time), activities of daily living and motor functioning (Unified Parkinson Disease Rating Scale part II and III, respectively), and freezing of gait (Freezing of Gait Questionnaire). These measures assess constructs which are different from, but somehow related to mobility capacity. For three correlations, the 95% confidence intervals crossed the hypothesized correlation borders.

Cronbach’s alpha (0.91) was within the proposed range of 0.70 to 0.95.^22^ Thus, the internal consistency reliability of the de Morton Mobility Index in people with Parkinson’s disease can be judged as excellent.

An intra-class correlation coefficient value of ⩾0.7 is considered sufficient for group comparisons, and a value of ⩾0.90 is an indicator of acceptable reliability for individual-level monitoring.^22,32^ In people with Parkinson’s disease, the inter-rater reliability of the de Morton Mobility Index (ICC = 0.88) was sufficiently high for group comparisons, and this reliability estimation is comparable to other inter-rater reliability estimations reported between 0.85 and 0.94.^8,13,33,34^ However, the de Morton Mobility Index seems limited for individual-level monitoring of mobility alterations over time if two assessors are involved. This interpretation is supported by the relatively large measurement error of 31% (limits of agreement) and 17.5 points (minimal detectable change with 90% confidence). The latter value is considerably higher than the minimal detectable change range of 6 to 10 points reported for older adults.^8,12,14,33,35^ A possible explanation could be the high level of heterogeneity in mobility in the included participants with Parkinson’s disease, indicated by the relatively large standard deviation of 22 points for the reliability sample (35% of the mean score). The standard error of measurement of 7.5 points, however, was acceptable^29^ and there was no item with absolute agreement <70%. Provided that all assessors carefully synchronize before clinical use, the de Morton Mobility Index can be reliably administered by different assessors in hospital patients with Parkinson’s disease.

This study provides evidence of the applicability of the de Morton Mobility Index over the whole mobility spectrum of individuals with Parkinson’s disease, since no floor or ceiling effects occurred at hospital admission. This result is in line with findings of Johnston et al.,^16^ who also reported no floor or ceiling effects in outpatient people with Parkinson’s disease.

The mean administration time of 6.6 minutes (range: 2–12) is comparable to other estimations of 5 to 10 minutes.^8,12,13,15^ Administration times for the de Morton Mobility Index of 5 to 10 minutes seem realistic in most individuals with Parkinson’s disease. High feasibility and short administration times of outcome measures facilitate routine clinical application and enlarge therapy time.

We used a combination of modern methods of latent trait theory (Rasch analysis) and methods of classical test theory to examine, for the first time, a broad set of measurement properties of the de Morton Mobility Index in a consecutive sample of hospital inpatients with Parkinson’s disease. The participants presented with a wide spectrum of disability, and most participants were in the moderate-to-severe disease stage (Hoehn and Yahr range from 1 to 5; 89% in stage 3 to 5). However, the external validity of this study might be limited since the data were collected from a single rehabilitation hospital only.

The sample size of 100 participants for the Rasch, construct validity, and internal consistency analyses seems sufficiently large^26,28^ and strengthens our findings. The size of the inter-rater reliability sample (*n* = 47) was slightly lower than intended.^21^

Stratford et al. recommended using data of stable patients to calculate measurement error over time.^27,31^ The calculation of minimal detectable change values performed in this study included the inter-rater variance and the participants’ intra-individual variance. Thus, the comparably large minimal detectable change value of 17.5 points might be biased and overestimated by the inter-rater variance included in the intra-class correlation coefficient value. Our minimal detectable change estimations should be considered with caution and verified by future studies, which should use test-retest reliability data of stable patients generated by a single assessor.

This study provides evidence of unidimensionality, structural and construct validity, internal consistency reliability, inter-rater reliability, and feasibility of the de Morton Mobility Index in hospital patients with Parkinson’s disease. The lack of any floor or ceiling effects on hospital admission indicates clinical value and applicability across the whole mobility spectrum. The de Morton Mobility Index can be administered without any special equipment, license charge, or long training period, and the administration time of the test is short. These advantages address some of the barriers to the use of measurement instruments,^36,37^ and may facilitate the application of this mobility measure in clinical care and research studies. Further research should focus on the measurement properties that are still unknown in people with Parkinson’s disease, such as responsiveness, minimal important change values, and prognostic validity. Since there are many measures available to measure mobility capacity of people with Parkinson’s disease, the psychometric quality and clinical utility of the de Morton Mobility Index should be compared to other measures in systematic reviews that follow recommended methods.^38^

Given consistently sufficient measurement properties of the de Morton Mobility Index’ across a variety of geriatric and neurological populations, this outcome assessment has the potential for clinical implementation, especially in clinical settings with (mixed) populations suffering from mobility limitations due to neurological and age-related conditions. In conclusion, the de Morton Mobility Index is a useful performance-based bedside test to measure mobility in hospital patients with Parkinson’s disease.

**Clinical messages**

- The de Morton Mobility Index is a constructually valid, reliable and unidimensional measure of mobility in hospital patients with Parkinson’s disease.
- Short administration times, no need for special equipment, simple and straightforward items together with an easy scoring system indicate high feasibility.

## Supplemental Material

Supp._1_assessments_format – Supplemental material for An investigation of the measurement properties of the de Morton Mobility Index for measuring mobility capacity in hospital patients with Parkinson’s disease Click here for additional data file. Supplemental material, Supp._1_assessments_format for An investigation of the measurement properties of the de Morton Mobility Index for measuring mobility capacity in hospital patients with Parkinson’s disease by Tobias Braun, Detlef Marks, Christian Thiel, Alexandra Menig and Christian Grüneberg in Clinical Rehabilitation

Supp._2_Methods_extended_format – Supplemental material for An investigation of the measurement properties of the de Morton Mobility Index for measuring mobility capacity in hospital patients with Parkinson’s disease Click here for additional data file. Supplemental material, Supp._2_Methods_extended_format for An investigation of the measurement properties of the de Morton Mobility Index for measuring mobility capacity in hospital patients with Parkinson’s disease by Tobias Braun, Detlef Marks, Christian Thiel, Alexandra Menig and Christian Grüneberg in Clinical Rehabilitation

Supp._3_Item_agreement_200327 – Supplemental material for An investigation of the measurement properties of the de Morton Mobility Index for measuring mobility capacity in hospital patients with Parkinson’s disease Click here for additional data file. Supplemental material, Supp._3_Item_agreement_200327 for An investigation of the measurement properties of the de Morton Mobility Index for measuring mobility capacity in hospital patients with Parkinson’s disease by Tobias Braun, Detlef Marks, Christian Thiel, Alexandra Menig and Christian Grüneberg in Clinical Rehabilitation

Supp._3_Item_agreement_200926_new – Supplemental material for An investigation of the measurement properties of the de Morton Mobility Index for measuring mobility capacity in hospital patients with Parkinson’s disease Click here for additional data file. Supplemental material, Supp._3_Item_agreement_200926_new for An investigation of the measurement properties of the de Morton Mobility Index for measuring mobility capacity in hospital patients with Parkinson’s disease by Tobias Braun, Detlef Marks, Christian Thiel, Alexandra Menig and Christian Grüneberg in Clinical Rehabilitation

Supp_figures – Supplemental material for An investigation of the measurement properties of the de Morton Mobility Index for measuring mobility capacity in hospital patients with Parkinson’s disease Click here for additional data file. Supplemental material, Supp_figures for An investigation of the measurement properties of the de Morton Mobility Index for measuring mobility capacity in hospital patients with Parkinson’s disease by Tobias Braun, Detlef Marks, Christian Thiel, Alexandra Menig and Christian Grüneberg in Clinical Rehabilitation

## References

[1] Keus S Munneke M Graziano M, et al. European physiotherapy guideline for Parkinson’s disease. Nijmegen: KNGF/ParkinsonNet, 2014.

[2] Sturkenboom I Thijssen MCE Gons-van Elsacker JJ, et al. Guidelines for occupational therapy in Parkinson’s disease rehabilitation. Nijmegen and Miami, FL: ParkinsonNet/NPF, 2012.

[3] Gor-Garcia-Fogeda MD La Cano de Cuerda R Carratala Tejada M, et al. Observational gait assessments in people with neurological disorders: a systematic review. Arch Phys Med Rehabil 2016; 97: 131–140. 26254954 10.1016/j.apmr.2015.07.018

[4] Bloem BR Marinus J Almeida Q, et al. Measurement instruments to assess posture, gait, and balance in Parkinson’s disease: critique and recommendations. Mov Disord 2016; 31: 1342–1355. 26945525 10.1002/mds.26572

[5] World Health Organization. International classification of functioning, disability and health: ICF. Geneva: World Health Organization, 2001.

[6] Rasch G. Probabilistic models for some intelligence and attainment tests. Expanded ed. Chicago, IL: University of Chicago Press, 1980.

[7] Tennant A Conaghan PG. The Rasch measurement model in rheumatology: what is it and why use it? When should it be applied, and what should one look for in a Rasch paper? Arthritis Rheum 2007; 57: 1358–1362. 18050173 10.1002/art.23108

[8] de Morton NA Davidson M Keating JL. The de Morton Mobility Index (DEMMI): an essential health index for an ageing world. Health Qual Life Outcomes 2008; 6: 63. 18713451 10.1186/1477-7525-6-63 PMC2551589

[9] de Morton NA Meyer C Moore KJ, et al. Validation of the de Morton Mobility Index (DEMMI) with older community care recipients. Australas J Ageing 2011; 30: 220–225. 22176568 10.1111/j.1741-6612.2010.00497.x

[10] de Morton NA Davidson M Keating J. Validity, responsiveness and the minimal clinically important difference for the de Morton Mobility Index (DEMMI) in an older acute medical population. BMC Geriatr 2010; 10: 72. 20920285 10.1186/1471-2318-10-72 PMC2958960

[11] Braun T Schulz R-J Hoffmann M, et al. German version of the de Morton Mobility Index. First clinical results from the process of the cross-cultural adaptation. Z Gerontol Geriatr 2015; 48: 154–163. 25388543 10.1007/s00391-014-0648-3

[12] Braun T Schulz R-J Reinke J, et al. Reliability and validity of the German translation of the de Morton Mobility Index (DEMMI) performed by physiotherapists in patients admitted to a sub-acute inpatient geriatric rehabilitation hospital. BMC Geriatr 2015; 15: 1660. 10.1186/s12877-015-0035-y PMC4424447 25935559

[13] Braun T Grüneberg C Thiel C, et al. Measuring mobility in older hospital patients with cognitive impairment using the de Morton Mobility Index. BMC Geriatr 2018; 18: 100. 29685107 10.1186/s12877-018-0780-9 PMC5913915

[14] Braun T Thiel C Schulz R-J, et al. Reliability of mobility measures in older medical patients with cognitive impairment. BMC Geriatr 2019; 19: 20. 30674278 10.1186/s12877-019-1036-z PMC6343264

[15] Braun T Marks D Thiel C, et al. Reliability and validity of the de Morton Mobility Index in individuals with sub-acute stroke. Disabil Rehabil 2019; 41: 1561–1570. 29397785 10.1080/09638288.2018.1430176

[16] Johnston M de Morton N Harding K, et al. Measuring mobility in patients living in the community with Parkinson disease. NeuroRehabilitation 2013; 32: 957–966. 23867421 10.3233/NRE-130919

[17] von Elm E Altman DG Egger M, et al. The strengthening the reporting of observational studies in epidemiology (STROBE) statement: guidelines for reporting observational studies. J Clin Epidemiol 2008; 61: 344–349. 18313558 10.1016/j.jclinepi.2007.11.008

[18] Kottner J Audige L Brorson S, et al. Guidelines for reporting reliability and agreement studies (GRRAS) were proposed. J Clin Epidemiol 2011; 64: 96–106. 21130355 10.1016/j.jclinepi.2010.03.002

[19] Mokkink LB de Vet HCW Prinsen CAC, et al. COSMIN risk of bias checklist for systematic reviews of patient-reported outcome measures. Qual Life Res 2018; 27: 1171–1179. 29260445 10.1007/s11136-017-1765-4 PMC5891552

[20] Hoehn MM Yahr MD. Parkinsonism: onset, progression and mortality. Neurology 1967; 17: 427–442. 6067254 10.1212/wnl.17.5.427

[21] Mokkink LB Terwee CB Patrick DL, et al. The COSMIN study reached international consensus on taxonomy, terminology, and definitions of measurement properties for health-related patient-reported outcomes. J Clin Epidemiol 2010; 63: 737–745. 20494804 10.1016/j.jclinepi.2010.02.006

[22] Terwee CB Bot SDM de Boer MR, et al. Quality criteria were proposed for measurement properties of health status questionnaires. J Clin Epidemiol 2007; 60: 34–42. 17161752 10.1016/j.jclinepi.2006.03.012

[23] Braun T Grüneberg C Coppers A, et al. Comparison of the de Morton Mobility Index and Hierarchical Assessment of Balance and Mobility in older acute medical patients. J Rehabil Med 2018; 50: 292–301. 29392333 10.2340/16501977-2320

[24] de Morton NA Harding KE Taylor NF, et al. Validity of the de Morton Mobility Index (DEMMI) for measuring the mobility of patients with hip fracture during rehabilitation. Disabil Rehabil 2013; 35: 105–111. 10.3109/09638288.2012.705220 22897700

[25] Pallant JF Tennant A. An introduction to the Rasch measurement model: an example using the Hospital Anxiety and Depression Scale (HADS). Br J Clin Psychol 2007; 46: 1–18. 17472198 10.1348/014466506x96931

[26] Linacre JM. Sample size and item calibration stability. Rasch Meas Trans 1994; 7: 328.

[27] de Vet HCW Terwee CB Mokkink LB, et al. Measurement in medicine: a practical guide. Cambridge and New York: Cambridge University Press, 2011.

[28] Terwee CB Mokkink LB Knol DL, et al. Rating the methodological quality in systematic reviews of studies on measurement properties: a scoring system for the COSMIN checklist. Qual Life Res 2012; 21: 651–657. 21732199 10.1007/s11136-011-9960-1 PMC3323819

[29] van Bloemendaal M Bout W Bus SA, et al. Validity and reproducibility of the Functional Gait Assessment in persons after stroke. Clin Rehabil 2019; 33: 94–103. 30084264 10.1177/0269215518791000

[30] Bland JM Altman DG. Statistical methods for assessing agreement between two methods of clinical measurement. Lancet 1986; 1: 307–310. 2868172

[31] Stratford PW Binkley JM Riddle DL. Health status measures: strategies and analytic methods for assessing change scores. Phys Ther 1996; 76: 1109–1123. 8863764 10.1093/ptj/76.10.1109

[32] Scientific Advisory Committee of the Medical Outcomes Trust. Assessing health status and quality-of-life instruments: attributes and review criteria. Qual Life Res 2002; 11: 193–205. 12074258 10.1023/a:1015291021312

[33] de Morton N Davidson M Keating JL. Reliability of the de Morton mobility index (DEMMI) in an older acute medical population. Physiother Res Int 2010; 16: 159–169. 21043046 10.1002/pri.493

[34] Jans MP Slootweg VC Boot CR, et al. Reproducibility and validity of the Dutch translation of the de Morton Mobility Index (DEMMI) used by physiotherapists in older patients with knee or hip osteoarthritis. Arch Phys Med Rehabil 2011; 92: 1892–1899. 22032224 10.1016/j.apmr.2011.05.011

[35] de Morton NA Lane K. Validity and reliability of the de Morton Mobility Index in the subacute hospital setting in a geriatric evaluation and management population. J Rehabil Med 2010; 42: 956–961. 21031293 10.2340/16501977-0626

[36] Duncan EAS Murray J. The barriers and facilitators to routine outcome measurement by allied health professionals in practice: a systematic review. BMC Health Serv Res 2012; 12: 96. 22506982 10.1186/1472-6963-12-96 PMC3358245

[37] Braun T Rieckmann A Weber F, et al. Current use of measurement instruments by physiotherapists working in Germany: a cross-sectional online survey. BMC Health Serv Res 2018; 18: 810. 30352584 10.1186/s12913-018-3563-2 PMC6199696

[38] Prinsen CAC Mokkink LB Bouter LM, et al. COSMIN guideline for systematic reviews of patient-reported outcome measures. Qual Life Res 2018; 27: 1147–1157. 29435801 10.1007/s11136-018-1798-3 PMC5891568

