# Supplementary material for: An investigation of the measurement properties of the de Morton Mobility Index for measuring mobility capacity in hospital patients with Parkinson’s disease
Source: Clin Rehabil. 2020 Nov 11;35(3):423–35. doi: 10.1177/0269215520966472 (PMC7944422; doi:10.1177/0269215520966472)
Supplement: Supp._1_assessments_format – Supplemental material for An investigation of the measurement properties of the de Morton Mobility Index for measuring mobility capacity in hospital patients with Parkinson’s disease [file Supp._1_assessments_format.pdf]

Supplementary file 1: Detailed description of the assessment procedures and comparator instruments

All assessments were performed in the participant's hospital room and on the ward. Instructions were given verbally. If necessary, and if this was allowed according to the formal instrument instructions, individual items or tasks were demonstrated by the assessor. Participants used the usual walking aid prescribed by the responsive physiotherapist. The same device was used for all assessments in a single session.

Similar items in different assessments were only performed once to reduce participant's burden, e.g. standing with both feet together is required in the de Morton Mobility Index (DEMMI), Performance Oriented Mobility Assessment (POMA) and the Berg Balance Scale (BBS). Breaks were offered between assessments. A hand-held digital stopwatch was used for all temporal outcomes. Walking distances were recorded with a digital measuring wheel. For participants requiring some kind of physical assistance during ambulation (Functional Ambulation Categories (FAC) scored ≤2), the walking tests were scored as "unable" (Timed Up And Go test (TUG), gait speed, 6 minute walk test (6minWT)) or "0" (POMA gait sub-scale), respectively. Stand-by assistance was provided for all participants whenever needed.

Most applied assessments (BBS, 5x chair rise test, TUG, gait speed, 6minWT, Unified Parkinson Disease Rating Scale) deem to be sufficiently valid for measuring mobility and physical functioning in pwPD as they are recommended by the European physiotherapy guideline for Parkinson's disease [1]. Validity of the other instruments is described individually.

Berg Balance Scale (BBS)

The BBS [2] is a psychometrically sound measure of balance for use in pwPD [3]. The patient's performance in 14 static and dynamic balance tasks is rated on an ordinal scale, with lower points indicating poorer balance.

Timed Up and Go test (TUG)

The TUG assesses basic mobility functions as it asks the patient to stand up from a chair, walk 3 m, turn around, walk back and return to the chair [4]. A familiarization trial was performed, followed by two counted trials, of which the mean (in sec) was used as the final TUG score. Shorter TUG times indicate higher mobility.

10 meter walk test

Fast gait speed (m/sec) was assessed over a distance of 10 m. Participants started 90 cm prior to the starting line and were timed from the moment their first foot crossed the starting line until their first foot crossed the finish line.

Functional Ambulation Categories (FAC)

The clinician-completed FAC distinguishes 6 levels of walking ability subjected to the amount of assistance required over a walking distance of 10 meters [5]. Lower scores, where physical assistance is needed, indicate poorer mobility than higher scores, where the patient is able to ambulate independently.

6-minute walk test (6minWT)

The 6minWT captures the distance in meters walked over a period of 6 minutes on a plain walkway [6]. It quantifies mobility and walking endurance. Longer distances indicate a better walking capacity and higher velocity. Breaks were offered if needed.

Performance Oriented Mobility Assessment (POMA)

Tinetti's POMA is a clinician-rated measure of mobility and fall risk, consisting of two sub-scales [7]. Ordinal scores are summed for the balance and the gait sub-scale. A total POMA score of 28 points indicates higher mobility. The POMA's reliability in individuals with PD is good, and there is evidence for construct validity [8].

5x chair rise test (5xCRT)

The 5xCRT is a quick measure of lower-extremity strength. We followed the protocol reported by Whitney et al. [9]. However, we stopped the time when the participant finished the fifth sit-to-stand transfer in a standing position.

Unified Parkinson Disease Rating Scale (UPDRS)

The UPDRS is a scale to assess PD-related disability and impairment [10, 11]. In this study, only results for the Part II (activities of daily living; 0 – 52 points) and Part III (motor functions; 0 – 108 points) components were available from medical records. Higher scores indicate more disability during activities of daily living and motor functioning. The UPDRS was scored by occupational therapy staff as part of usual care. We only used UPDRS scores from participants who administered the UPDRS within 7 days after admission.

Freezing of Gait Questionnaire (FOGQ)

The FOGQ is a 6-item patient-reported outcome measure to assess freezing of gait in pwPD [12, 13]. The scoring system ranges from 0 to 24 points. The higher the score is, the more the freezing of gait is pronounced.

Functional Independence Measure (FIM)

The FIM is one of the most established generic disability measures [14]. On an ordinal scale, composed of 18 items, a patient's independence in several activities is each rated on a 7 point Likert scale ranging from 1 (total dependence) to 7 (total independence). Higher scores indicate better functioning. In this study, the total score as well as the mobility subscale were used. The latter included the following 5 items: bed to chair transfer, toilet transfer, shower transfer, locomotion, stairs. Therefore, the highest achievable FIM mobility subscale score was 35 points. The FIM was administered as part of usual care by the nursing staff.

References Supplementary file 2

1. Keus S, Munneke M, Graziano M, Paltamaa J, Pelosin E, Domingos J, et al. European physiotherapy guideline for Parkinson's disease. The Netherlands: KNGF/ParkinsonNet; 2014.
2. Berg KO, Wood-Dauphinee SL, Williams JI, Maki B. Measuring balance in the elderly: validation of an instrument. Can J Public Health. 1992;83 Suppl 2:11.
3. Qutubuddin AA, Pegg PO, Cifu DX, Brown R, McNamee S, Carne W. Validating the Berg Balance Scale for patients with Parkinson's disease: a key to rehabilitation evaluation. Arch Phys Med Rehabil. 2005;86:789–92. doi:10.1016/j.apmr.2004.11.005.
4. Podsiadlo D, Richardson S. The timed "Up & Go": a test of basic functional mobility for frail elderly persons. J Am Geriatr Soc. 1991;39:142–8.
5. Holden MK, Gill KM, Magliozzi MR, Nathan J, Piehl-Baker L. Clinical gait assessment in the neurologically impaired. Reliability and meaningfulness. Phys Ther. 1984;64:35–40.
6. Enright PL, McBurnie MA, Bittner V, Tracy RP, McNamara R, Arnold A, Newman AB. The 6-min walk test: a quick measure of functional status in elderly adults. Chest. 2003;123:387–98.
7. Tinetti ME. Performance-oriented assessment of mobility problems in elderly patients. J Am Geriatr Soc. 1986;34:119–26.
8. Kegelmeyer DA, Kloos AD, Thomas KM, Kostyk SK. Reliability and validity of the Tinetti Mobility Test for individuals with Parkinson disease. Phys Ther. 2007;87:1369–78. doi:10.2522/ptj.20070007.
9. Whitney SL, Wrisley DM, Marchetti GF, Gee MA, Redfern MS, Furman JM. Clinical measurement of sit-to-stand performance in people with balance disorders: validity of data for the Five-Times-Sit-to-Stand Test. Phys Ther. 2005;85:1034–45.

10. Fahn S, Elton RL. Unified Parkinson’s disease rating scale. In: Fahn S, Marsden CD, Calne D, Goldstein M(e), editors. Recent developments in Parkinson’s disease. Florham Park, NJ: Macmillan Health Care Information; 1987. p. 153.
11. Movement Disorder Society Task Force on Rating Scales for Parkinson’s Disease. The Unified Parkinson’s Disease Rating Scale (UPDRS): status and recommendations. Mov Disord. 2003;18:738–50. doi:10.1002/mds.10473.
12. Giladi, Shabtai, Simon, Biran, Tal, Korczyn. Construction of freezing of gait questionnaire for patients with Parkinsonism. Parkinsonism Relat. Disord. 2000;6:165–70.
13. Vogler A, Janssens J, Nyffeler T, Bohlhalter S, Vanbellingen T. German translation and validation of the “freezing of gait questionnaire” in patients with Parkinson’s disease. Parkinsons Dis. 2015;2015:982058. doi:10.1155/2015/982058.
14. Keith RA, Granger CV, Hamilton BB, Sherwin FS. The functional independence measure: a new tool for rehabilitation. Adv Clin Rehabil. 1987;1:6–18.
