# Supplementary material for: An investigation of the measurement properties of the de Morton Mobility Index for measuring mobility capacity in hospital patients with Parkinson’s disease
Source: Clin Rehabil. 2020 Nov 11;35(3):423–35. doi: 10.1177/0269215520966472 (PMC7944422; doi:10.1177/0269215520966472)
Supplement: Supp._2_Methods_extended_format – Supplemental material for An investigation of the measurement properties of the de Morton Mobility Index for measuring mobility capacity in hospital patients with Parkinson’s disease [file Supp._2_Methods_extended_format.pdf]

Supplementary file 2: Additional information on the statistical analyses

Criteria for the Rasch analysis

The unrestricted (partial credit) Rasch polytomous model was used with a conditional pair-wise parameter estimation and two class intervals. Overall fit of data to the model is deemed acceptable if the following criteria are fulfilled (adopted from Mills et al. [1]):

(1) Both total chi-square probability and individual item chi-square probability values non-significant.
(2) Individual item fit residual, by convention, within ±2.5.
(3) Mean and standard deviation of both item fit residual and person fit residual approaching 0 and 1, respectively.
(4) Ordered item category thresholds.
(5) Person-item separation index (PSI) (reliability) greater than 0.70 for group use and 0.85 for individual use.
(6) Unidimensionality (all items reflecting a single underlying latent trait) by independent t-test at the person abilities showing less than 5% of tests to be significant or the lower bound of a binomial 95% confidence interval of the observed proportion overlaps 5% [2].
(7) Pearson correlation coefficients between item residuals between between 0.2 and 0.3 above the average of all item residual correlations (local independence) [3]. If the correlation was between 0.2 and 0.3 above the average, a subtest analysis using the correlated items was undertaken. Local dependence was considered if the internal consistency (PSI) of the whole item set differed substantially from the PSI in the subtest.
(8) Differential Item Functioning (DIF) occurs when different groups within the sample (e.g., women and men) respond in a different manner to an individual item, despite equal levels of the underlying characteristic being measured. ANOVA probability for differential item functioning (DIF) non-significant (5% alpha with Bonferroni correction) for the following factors: sex (male and female), age (split at median: 18 to 72 years and 73+ years) and cognitive impairment by MMSE (split at median: 0 to 27 and 28 to 30 points). This is undertaken with a two way ANOVA with class interval (grouped level of mobility) and the external factor (e.g. sex) as main effects. Uniform DIF is then for the main effect of the factor (e.g. gender; and there is another for class interval) and non-uniform DIF is the interaction between class interval and the factor. For DIF analysis, clinical meaningful groups of approximately equal sizes are needed.

Construct validity

Formulated hypotheses:

- H1 – H3: De Morton Mobility Index (DEMMI) scores correlate strongly (>0.7) with other broad measures of mobility (Performance Oriented Mobility Assessment, Timed Up and Go test, mobility subscale of the Functional Independence Measure (FIM)), as reported for people with Parkinson's disease (PD) [4], people with stroke [5], and older people [6–9].
- H4: DEMMI scores correlate strongly (>0.7) with the 6-minute walk test, a measure of walking endurance. Others reported Spearman's rho correlations between DEMMI and 2- or 6-minute walk test of 0.76 [10], 0.70 [9] and 0.76 [11] in older hospital patients. In people with stroke, the correlation was 0.91 [5].
- H5: DEMMI scores correlate strongly (>0.7) with the Functional Ambulation Categories (FAC), a measure of ambulation. Others reported Spearman's rho correlations between DEMMI and FAC of 0.92 [9], 0.87 [11] in older people and 0.93 in people with stroke [5].
- H6: DEMMI scores correlate strongly (>0.7) with the Berg Balance Scale, a measure of balance. In people with PD and stroke, correlations of 0.84 and 0.96 have been reported, respectively [4, 5].
- H7: DEMMI scores correlate moderately ($0.5 < \text{rho} \leq 0.7$) with gait speed, a single component mobility measure. Johnston et al. [4] reported rho = 0.57 between DEMMI scores and 6-meter gait speed in people with PD.
- H8: DEMMI scores correlate moderately ($-0.7 \leq \text{rho} < -0.5$) with the 5x chair rise test, a single component mobility measures. Jans et al. [7] and Braun et al. [9] both reported rho = -0.63 between DEMMI and 5x chair rise time in older patients with knee or hip osteoarthritis and with cognitive impairment, respectively.
- H9 – H10: DEMMI scores correlate moderately with part II of the Unified Parkinson Disease Rating Scale (UPDRS; $-0.7 \leq \text{rho} < -0.5$) and the FIM total score ($0.5 < \text{rho} \leq 0.7$), both measures of functional independence, disability and activities of daily living (ADL). De Morton et al. [6] and Braun et al. [5] reported correlations between the DEMMI and ADL disability of 0.68 (Barthel Index; older people) and 0.85 (FIM, people with stroke), respectively.
- H11: DEMMI scores correlate moderately ($-0.7 \leq \text{rho} < -0.5$) with part III of the UPDRS, a measure of motor functioning in people with PD. No comparison between both scales has been reported. Some items of the UPDRS deal with mobility and lower limb functioning, but some items assess upper limb and trunk motor functions. Thus, we consider part III of the UPDRS to measure a related (motor functioning), but not similar construct (mobility) then the DEMMI.

- H12: DEMMI scores correlate moderately (-0.7 ≤ rho < -0.5) with the Freezing of Gait Questionnaire (FOGQ). Vogler et al. [12] reported a correlation of 0.52 between the FOGQ and the mobility subscale of the Parkinson Disease Questionnaire 39 [13].
- H13: Ambulatory participants (FAC ≥ 3) ambulating without a walking aid have higher DEMMI scores than participants using a walking aid.
- H14: Independent walkers (FAC ≥ 4) have higher DEMMI scores than non-ambulatory participants or dependent walkers (FAC ≤ 3).
- H15: Participants with mild-moderate PD symptoms (Hoehn & Yahr stage 1 – 3) have higher DEMMI scores than participants with severe symptoms (Hoehn & Yahr stage 4 – 5).

We applied one-tailed Spearman's rho analyses because directions of the correlations were hypothesized *a priori*. A one-sided Mann Whitney U test for independent samples was used to compare groups as hypotheses were formulated *a priori*. For each hypothesis on known-groups validity, we expected a statistically significant difference between the DEMMI mean scores of both groups.

Between the DEMMI and the outcomes, in which lower scores represent better functioning (TUG, 5xCRT, UPDRS, FOGQ), a negative correlation was hypothesized. The expected strengths of correlations are always reported unidirectional to improve readability.

We decided against defining an a priori threshold of e.g. ≥75% of hypotheses which need to be confirmed in order for a measurement instrument to be valid [14, 15]. Along with others [16], we do not think that the broad concept of construct validity can be judged as "good" or "bad" according to an arbitrary threshold of confirmed hypotheses of varying importance. Instead, we leave it to the reader to decide which percentage of confirmed hypotheses is deemed acceptable.

Inter-rater reliability

The $ICC_{AGREEMENT}$ was calculated by dividing the systematic differences between the ''true'' scores of participants by the error variance consisting of the systematic differences between the ''true'' scores of participants, the variance due to systematic differences between raters and the residual variance [17].

Measurement error

The standard error of measurement ($SEM_{AGREEMENT}$) was calculated using the same variance components used for the $ICC_{AGREEMENT}$ calculation and by taking the square root of the variance between the raters and the error variance of the $ICC_{AGREEMENT}$ [17]. The SEM was satisfactory if it was ≤10% of the total scale range (100 DEMMI points) [18].

Interpretability: Limits of agreement/Bland and Altman plot

The method of Bland and Altman was used to illustrate agreement between two raters [19]. The 95% limits of agreement require homoscedasticity and normally distributed differences [20]. A positive Kendall's tau (τ) correlation between the absolute differences and the corresponding means [21] >0.1 was deemed to denote heteroscedasticity. In case of heteroscedastic data, the following formula was used to calculate the limits of agreement: $-2X\,\frac{(10^a-1)}{(10^a+1)}\ and + 2X\,\frac{(10^a-1)}{(10^a+1)}$, with a = 95% limits of agreement of the 10log transformed data and X the mean score [22].

Interpretability: Minimal Detectable Change

The minimal detectable change (MDC) with 90% and 95% confidence, a quantification of absolute agreement, was calculated based on the inter-rater reliability data as $MDC_{90}=1.64*\sqrt{2}*SEM_{AGREEMENT}$ and $MDC_{95}=1.96*\sqrt{2}*SEM_{AGREEMENT}$, respectively. The MDC_{95} (MDC_{90}, respectively) is defined as the minimal amount of change that needs to occur between repeated assessments in an individual to exceed, with 95% (90%) confidence, the error of the measurement [23].

References Supplementary file 2

1. Mills RJ, Pallant JF, Koufali M, Sharma A, Day S, Tennant A, Young CA. Validation of the Neurological Fatigue Index for stroke (NFI-Stroke). Health Qual Life Outcomes. 2012;10:51.
2. Tennant A, Pallant JF. Unidimensionality Matters! (A Tale of Two Smiths?). Rasch Measurement Transactions. 2006;20:1048–51.
3. Christensen KB, Makransky G, Horton M. Critical Values for Yen's Q3: Identification of Local Dependence in the Rasch Model Using Residual Correlations. Applied Psychological Measurement. 2017;41:178–94.
4. Johnston M, Morton N de, Harding K, Taylor N. Measuring mobility in patients living in the community with Parkinson disease. NeuroRehabilitation. 2013;32:957–66.
5. Braun T, Marks D, Thiel C, Grüneberg C. Reliability and validity of the de Morton Mobility Index in individuals with sub-acute stroke. Disabil Rehabil. 2018:Epub ahead of print.
6. de Morton NA, Davidson M, Keating JL. The de Morton Mobility Index (DEMMI): an essential health index for an ageing world. Health Qual Life Outcomes. 2008;6:63.
7. Jans MP, Slootweg VC, Boot CR, de Morton NA, van der Sluis G, van Meeteren NL. Reproducibility and validity of the Dutch translation of the de Morton Mobility Index (DEMMI) used by physiotherapists in older patients with knee or hip osteoarthritis. Arch Phys Med Rehabil. 2011;92:1892–9.

8. Braun T, Schulz R-J, Reinke J, van Meeteren NL, Morton NA de, Davidson M, et al. Reliability and validity of the German translation of the de Morton Mobility Index (DEMMI) performed by physiotherapists in patients admitted to a sub-acute inpatient geriatric rehabilitation hospital. BMC Geriatr. 2015;15:1660.
9. Braun T, Grüneberg C, Thiel C, Schulz R-J. Measuring mobility in older hospital patients with cognitive impairment using the de Morton Mobility Index. BMC Geriatr. 2018;18:100.
10. de Morton NA, Harding KE, Taylor NF, Harrison G. Validity of the de Morton Mobility Index (DEMMI) for measuring the mobility of patients with hip fracture during rehabilitation. Disabil Rehabil. 2013;35:105–11.
11. Braun T, Grüneberg C, Coppers A, Tofaute L, Thiel C. Comparison of the de Morton Mobility Index and Hierarchical Assessment of Balance and Mobility in older acute medical patients. J Rehabil Med. 2018;50:292-301.
12. Vogler A, Janssens J, Nyffeler T, Bohlhalter S, Vanbellingen T. German translation and validation of the "freezing of gait questionnaire" in patients with Parkinson's disease. Parkinsons Dis. 2015;2015:982058.
13. Peto V, Jenkinson C, Fitzpatrick R. PDQ-39: A review of the development, validation and application of a Parkinson's disease quality of life questionnaire and its associated measures. J Neurol. 1998;245:S10-S14.
14. Terwee CB, Bot SDM, de Boer MR, van der Windt DAWM, Knol DL, Dekker J, et al. Quality criteria were proposed for measurement properties of health status questionnaires. J Clin Epidemiol. 2007;60:34–42.
15. Prinsen CAC, Mokkink LB, Bouter LM, Alonso J, Patrick DL, Vet HCW de, Terwee CB. COSMIN guideline for systematic reviews of patient-reported outcome measures. Qual Life Res. 2018;27:1147–57.
16. Reeve BB, Wyrwich KW, Wu AW, Velikova G, Terwee CB, Snyder CF, et al. ISOQOL recommends minimum standards for patient-reported outcome measures used in patient-centered outcomes and comparative effectiveness research. Qual Life Res. 2013;22:1889–905.
17. de Vet HCW, Terwee CB, Mokkink LB, Knol DL. Measurement in medicine: A practical guide. Cambridge, New York: Cambridge University Press; 2011.
18. van Bloemendaal M, Bout W, Bus SA, Nollet F, Geurts AC, Beelen A. Validity and reproducibility of the Functional Gait Assessment in persons after stroke. Clinical Rehabilitation. 2018:269215518791000.
19. Bland JM, Altman DG. Statistical methods for assessing agreement between two methods of clinical measurement. Lancet. 1986;1:307–10.

20. Altman DG, Bland JM. Measurement in Medicine: The Analysis of Method Comparison Studies. The Statistician. 1983;32:307–17.
21. Brehm MA, Scholtes VA, Dallmeijer AJ, Twisk JW, Harlaar J. The importance of addressing heteroscedasticity in the reliability analysis of ratio-scaled variables: An example based on walking energy-cost measurements. Dev Med Child Neurol. 2012;54:267–73.
22. Euser AM, Dekker FW, Le Cessie S. A practical approach to Bland-Altman plots and variation coefficients for log transformed variables. Journal of Clinical Epidemiology. 2008;61:978–82.
23. Stratford PW, Binkley JM, Riddle DL. Health status measures: strategies and analytic methods for assessing change scores. Phys Ther. 1996;76:1109–23.
