# Supplementary material for: An investigation of the measurement properties of the de Morton Mobility Index for measuring mobility capacity in hospital patients with Parkinson’s disease
Source: Clin Rehabil. 2020 Nov 11;35(3):423–35. doi: 10.1177/0269215520966472 (PMC7944422; doi:10.1177/0269215520966472)

Supplementary file 3:

Agreement between the two raters per DEMMI item

No.	Item	Agreement (%) with 95% CI	Kappa (ķ) with 95% CI
1	Bridge	91 (79 – 97)	0.62 (0.30 – 0.95)
2	Roll onto side	91 (79 – 97)	0.62 (0.28 – 0.96)
3	Lying to sitting	83 (69 – 92)	0.47 (0.09 – 0.84)
4	Sit unsupported in chair	100 (91 – 100)	1.00
5	Sit to stand from chair	94 (81 – 98)	0.75 (0.44 – 1.00)
6	Sit to stand without using arms	85 (71 – 93)	0.62 (0.37 – 0.87)
7	Stand unsupported	98 (87 – 100)	0.79 (0.39 – 1.00)
8	Stand feet together	81 (66 – 90)	0.43 (0.14 – 0.72)
9	Stand on toes	72 (57 – 84)	0.30 (0 – 0.59)
10	Tandem stand with eyes closed	85 (71 – 93)	0.38 (0 – 0.75)
11	Walking distance	91 (79 – 97)	0.52 (0.10 – 0.94)
12	Walking independence	85 (71 – 93)	0.82 (0.69 – 0.94)
13	Pick up pen from floor	91 (78 – 97)	0.67 (0.36 – 0.97)
14	Walk 4 steps backwards	85 (71 – 93)	0.51 (0.20 – 0.81)
15	Jump	87 (74 – 95)	0.75 (0.56 – 0.93)

Histograms of de Morton Mobility Index scores by sample

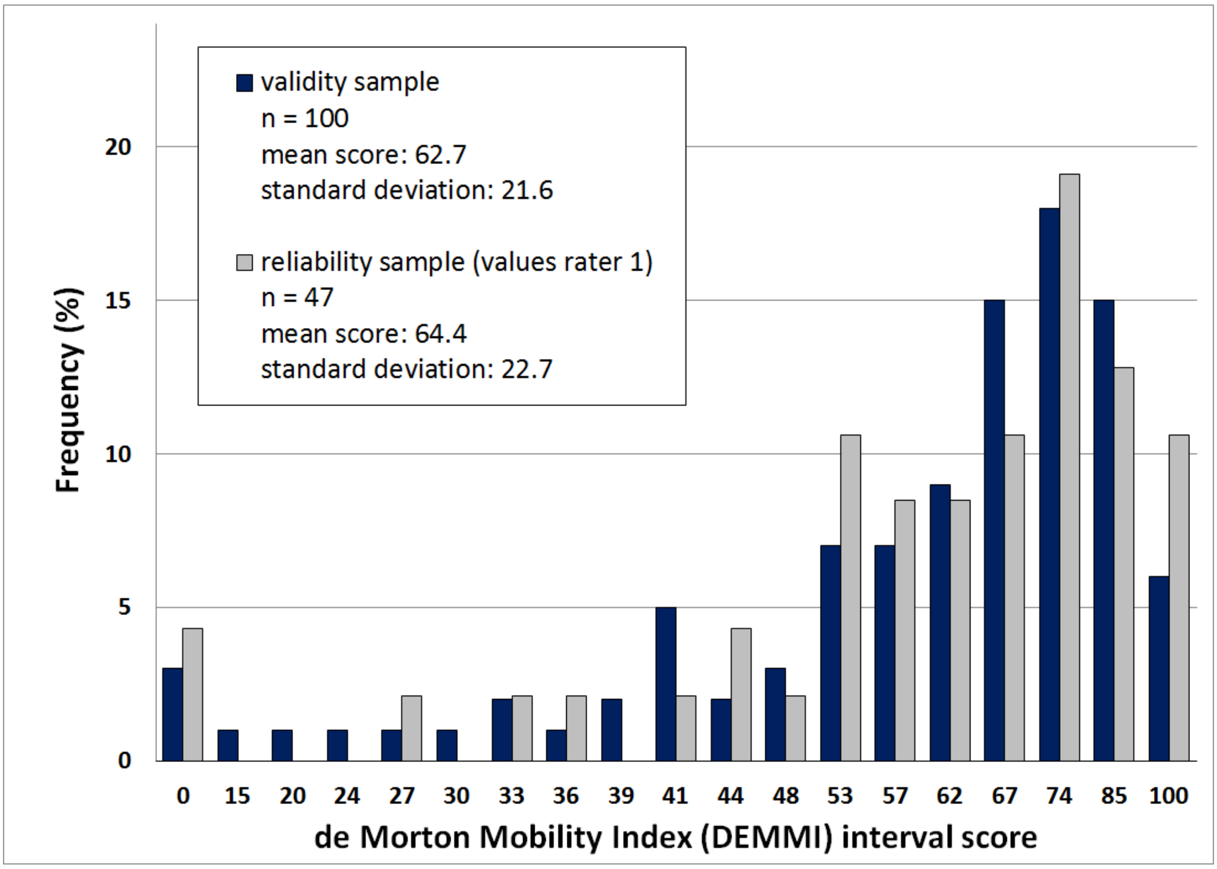

Absolute agreement per item of the de Morton Mobility Index (DEMMI)

No.	DEMMI Item	Percent (95% CI)
1	Bridge	91 (79 – 97)
2	Roll onto side	91 (79 – 97)
3	Lying to sitting	83 (69 – 92)
4	Sit unsupported in chair	100 (91 – 100)
5	Sit to stand from chair	94 (81 – 98)
6	Sit to stand without using arms	85 (71 – 93)
7	Stand unsupported	98 (87 – 100)
8	Stand feet together	81 (66 – 90)
9	Stand on toes	72 (57 – 84)
10	Tandem stand with eyes closed	85 (71 – 93)
11	Walking distance	91 (79 – 97)
12	Walking independence	85 (71 – 93)
13	Pick up pen from floor	91 (78 – 97)
14	Walk 4 steps backwards	85 (71 – 93)
15	Jump	87 (74 – 95)

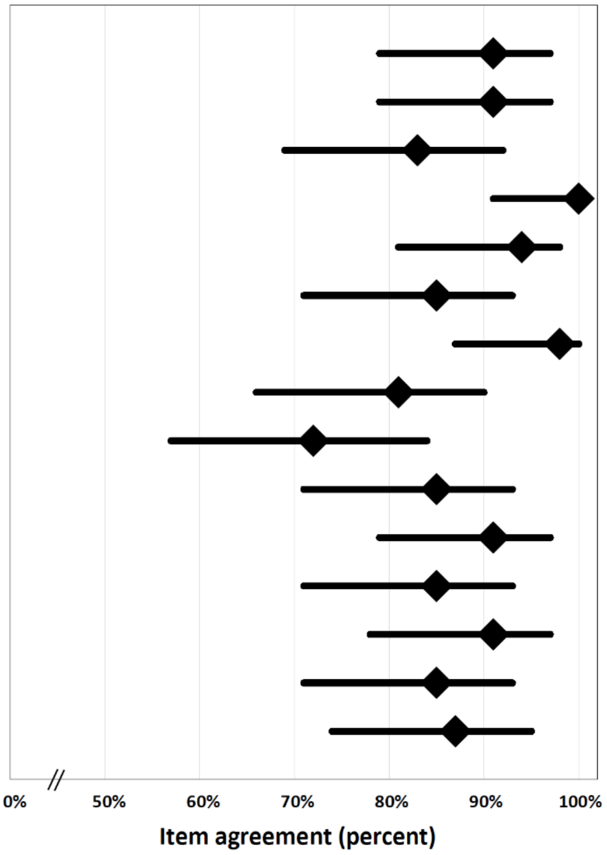

Supplement: Supp._3_Item_agreement_200926_new – Supplemental material for An investigation of the measurement properties of the de Morton Mobility Index for measuring mobility capacity in hospital patients with Parkinson’s disease [file Supp._3_Item_agreement_200926_new.pdf]
