# Supplementary material for: An investigation of the measurement properties of the de Morton Mobility Index for measuring mobility capacity in hospital patients with Parkinson’s disease
Source: Clin Rehabil. 2020 Nov 11;35(3):423–35. doi: 10.1177/0269215520966472 (PMC7944422; doi:10.1177/0269215520966472)

Supplemental Figure 1: Histograms of de Morton Mobility Index scores by sample

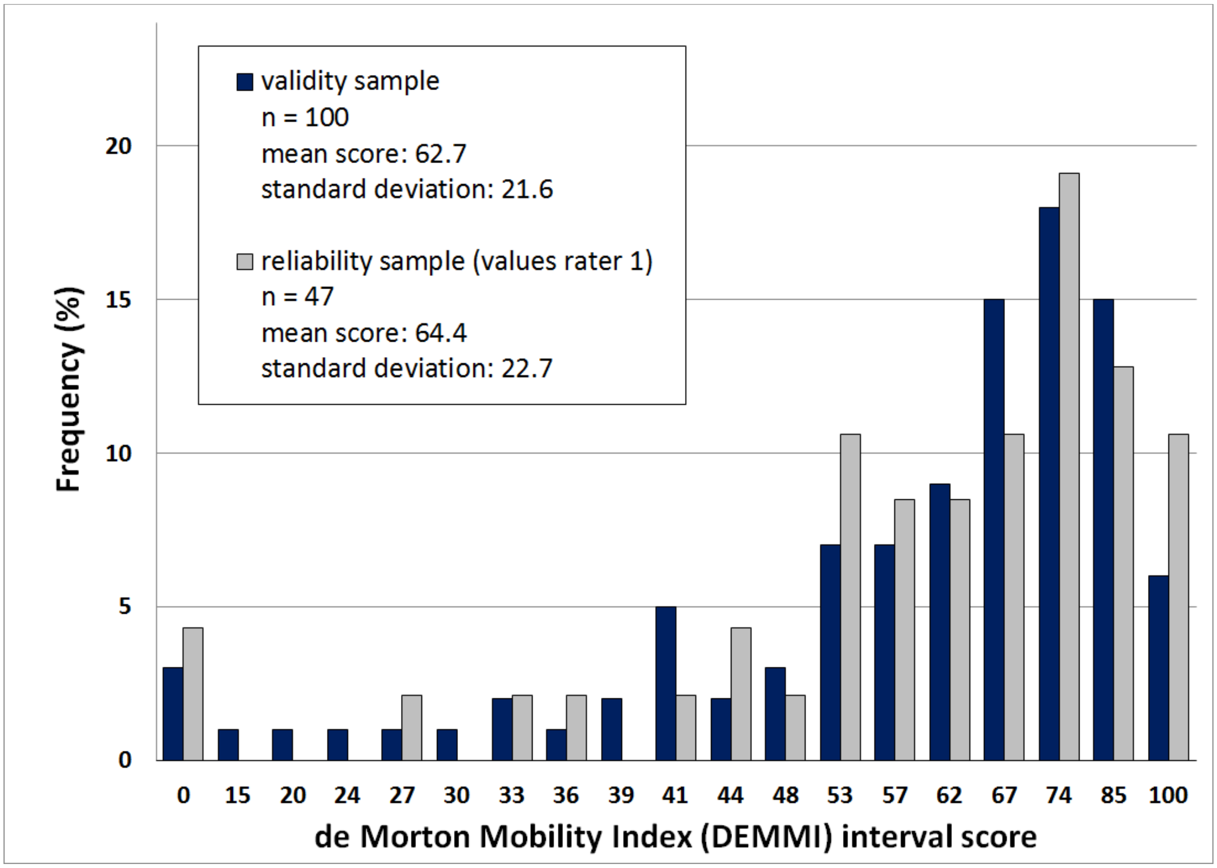

Supplemental Figure 2: Absolute agreement per item of the de Morton Mobility Index (DEMMI)

No.	DEMMI Item	Percent (95% CI)
1	Bridge	91 (79 – 97)
2	Roll onto side	91 (79 – 97)
3	Lying to sitting	83 (69 – 92)
4	Sit unsupported in chair	100 (91 – 100)
5	Sit to stand from chair	94 (81 – 98)
6	Sit to stand without using arms	85 (71 – 93)
7	Stand unsupported	98 (87 – 100)
8	Stand feet together	81 (66 – 90)
9	Stand on toes	72 (57 – 84)
10	Tandem stand with eyes closed	85 (71 – 93)
11	Walking distance	91 (79 – 97)
12	Walking independence	85 (71 – 93)
13	Pick up pen from floor	91 (78 – 97)
14	Walk 4 steps backwards	85 (71 – 93)
15	Jump	87 (74 – 95)

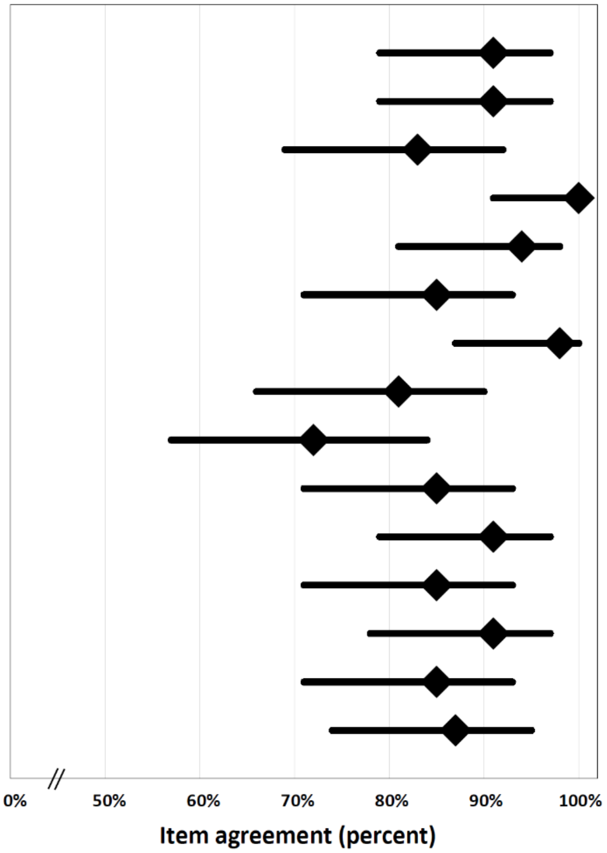

Supplement: Supp_figures – Supplemental material for An investigation of the measurement properties of the de Morton Mobility Index for measuring mobility capacity in hospital patients with Parkinson’s disease [file Supp_figures.pdf]
